# Defining the risk landscape in the context of pathogen pollution: *Toxoplasma gondii* in sea otters along the Pacific Rim

**DOI:** 10.1098/rsos.171178

**Published:** 2018-07-04

**Authors:** Tristan L. Burgess, M. Tim Tinker, Melissa A. Miller, James L. Bodkin, Michael J. Murray, Justin A. Saarinen, Linda M. Nichol, Shawn Larson, Patricia A. Conrad, Christine K. Johnson

**Affiliations:** 1 Karen C Drayer Wildlife Health Center, One Health Institute, 1089 Veterinary Medicine Drive, University of California, Davis, CA 95616, USA; 2 US Geological Survey, Western Ecological Research Center, Long Marine Laboratory, 100 Shaffer Road, Santa Cruz, CA, 95060, USA; 3 Marine Wildlife Veterinary Care and Research Center, California Department of Fish and Wildlife, Santa Cruz, CA, 95060, USA; 4 US Geological Survey, Alaska Science Center, 4201 University Drive, Anchorage, AK, 99503, USA; 5 Monterey Bay Aquarium, 886 Cannery Row, Monterey, CA, 93940, USA; 6 New College of Florida, 5800 Bay Shore Road, Sarasota, FL 34243, USA; 7 Fisheries and Oceans Canada, Pacific Biological Station, 3190 Hammond Bay Road, Nanaimo, British Columbia, Canada V9T 6N7; 8 The Seattle Aquarium, 1483 Alaskan Way, Pier 59, Seattle, WA 98101, USA

**Keywords:** *Enhydra lutris*, pathogen movement, anthropogenic land use, landscape change, spatial scale, *Toxoplasma gondii*

## Abstract

Pathogens entering the marine environment as pollutants exhibit a spatial signature driven by their transport mechanisms. The sea otter (*Enhydra lutris*), a marine animal which lives much of its life within sight of land, presents a unique opportunity to understand land–sea pathogen transmission. Using a dataset on *Toxoplasma gondii* prevalence across sea otter range from Alaska to California, we found that the dominant drivers of infection risk vary depending upon the spatial scale of analysis. At the population level, regions with high *T. gondii* prevalence had higher human population density and a greater proportion of human-dominated land uses, suggesting a strong role for population density of the felid definitive host of this parasite. This relationship persisted when a subset of data were analysed at the individual level: large-scale patterns in sea otter *T. gondii* infection prevalence were largely explained by individual exposure to areas of high human housing unit density, and other landscape features associated with anthropogenic land use, such as impervious surfaces and cropping land. These results contrast with the small-scale, within-region analysis, in which age, sex and prey choice accounted for most of the variation in infection risk, and terrestrial environmental features provided little variation to help in explaining observed patterns. These results underscore the importance of spatial scale in study design when quantifying both individual-level risk factors and landscape-scale variation in infection risk.

## 1. Background

Marine pathogen pollution involves the transport of potentially pathogenic terrestrial-based microorganisms to the ocean, either directly by flows of water or air, or indirectly by mobile intermediate or transport hosts [1]. The input of pathogens from the terrestrial environment may drive spatial patterns in the incidence of infection with pollutant pathogens, particularly in the absence of secondary horizontal transmission within the marine host species. During the past two decades, much effort has been devoted to the study of *Toxoplasma gondii* in sea otters (*Enhydra lutris*)*—*partly because this pollutant pathogen causes protozoal encephalitis in a threatened marine mammal species [2]—but also because toxoplasmosis in sea otters represents a model system for elucidating the mechanisms underlying marine pathogen pollution [3–5]. The sea otter as a host species, and this study system more generally, have several features that lend themselves to understanding marine pathogen pollution. Firstly sea otters, especially females, exhibit marked site fidelity [6], and so the nature of their habitat and exposures can be predicted with some accuracy based on capture locations. Secondly, these animals also live their whole lives near shore and bring all prey items to the surface to process, which facilitates accurate observation of diet and habitat use [6]. Finally, *T. gondii* infects only endothermic organisms, whereas sea otters prey almost exclusively on ectotherms [7], and so are infected by ingestion of infectious oocysts, either directly via contaminated water or when contained within invertebrate transport hosts. Since the sea otter lives permanently outside of its thermoneutral zone [8] it consumes large volumes of generally sessile invertebrate prey, effectively contacting any pathogens contained in that prey in the process.

Major advances have been made in understanding the basic epidemiology of *T. gondii* in the near shore environment, and in determining individual-level risk factors for infection in southern sea otters (*E. lutris nereis*). Previous work has identified individual-level intrinsic and behavioural features that increase the likelihood of *T. gondii* infection in this marine mammal host, including increasing age, male sex [9] and consumption of a diet high in marine snails [10]. Terrestrial felids (both wild and domestic) are the only definitive (oocyst-shedding) hosts of *T. gondii* [11]. Recent studies on the shedding of infectious oocysts by terrestrial felids [12] have provided further insight into the epidemiology and ecology of this pathogen on land, but less is known about features of the terrestrial environment that influence the spatial variation in infection risk for sea otters. While specific high risk locations along the California coastline have been previously identified, the only general feature of the terrestrial environment associated with a higher risk of *T. gondii* infection in sea otters established to date is proximity to (and magnitude of) sources of freshwater runoff [4]. Domestic cat population density has been shown to be associated with human population density and development-related environmental disturbance [13–15]. Furthermore, vegetation and land cover can directly affect the likelihood of oocysts entering freshwater or being filtered out by wetlands [16–18].

We hypothesize that the risk of exposure to a specific pollutant pathogen can be understood as a combination of (i) the distribution and concentration of pollutant pathogens in the host's habitat—i.e. the ‘risk landscape', and (ii) the individual intrinsic and behavioural features of the host that determine its interaction with the risk landscape. On this basis, we hypothesize that variation in human development, population density and land use together explain substantial variation in *T. gondii* oocyst concentration in freshwater runoff, and hence, the larger scale spatial variation in the infection risk landscape for sea otters. To test these hypotheses, we examined the prevalence of *T. gondii* infection, based on serological testing, in sea otters along the North American Pacific coast from Alaska to California, and evaluated the influence of key landscape-scale drivers in coastal watersheds within the context of three independent analyses with very different spatial scales: (i) a conventional local-scale (within-region) analysis of individual, behavioural and landscape-derived risk factors over a small spatial extent within California; (ii) a coarse-grained analysis among regions, comparing regional average values of terrestrial risk factors and mean regional *T. gondii* prevalence; and (iii) a fine-grained analysis covering a broad study area. The third analysis combines within- and among-region approaches to evaluate estimated individual-level exposure to terrestrial factors derived from freshwater runoff. Understanding key drivers of the risk landscape (in addition to individual risk factors) is particularly important because it is comparatively more likely to be influenced by human activity, and in a wild animal species is potentially more amenable to mitigation measures.

## 2. Results

### 2.1. Within-region drivers of infection risk

To examine within-region drivers of infection risk, a small-scale analysis examined data on *T. gondii* infection status, age, sex, diet and indices of human population and land cover from a group of 131 animals in three areas of intensive observational study in California—Monterey Bay, Monterey Peninsula and the Big Sur coast. The ‘best fit' logistic regression model (based on Akaike information criteria (AIC); table 1) included only male sex (odds ratio (OR) = 2.62, *p* = 0.066) and consumption of a diet including greater than 10% snail biomass (OR = 5.10, *p* = 0.002). Despite noticeable variation in some land cover variables (e.g. developed land, forest) and population density of watersheds ranging from 0.38 to 900 persons km^−2^ within this smaller study area, no significant within-region associations with land cover types or human population were statistically significant after accounting for individual-level intrinsic and dietary variables.

**Table 1.** Within-region analysis. (Multivariate logistic regression model predicting *T. gondii* prevalence among 131 live captured sea otters (1998–2013) at Monterey Bay, Monterey Peninsula and Big Sur (California). OR, odds ratio; 95% CI, 95% confidence interval.)

variable	level	OR	95% CI	*p*-value
sex	female	1.00	—	REF
	male	2.62	(0.94–7.29)	0.066
snail consumption	>10% biomass	5.10	(1.8–14.41)	0.002

### 2.2. Among-region drivers of infection risk

Anticipating large differences in sea otter *T. gondii* infection prevalence among regions, we hypothesized that prevalence would be associated with land use/land cover and human population density. To understand the factors driving the risk landscape, a large-scale (among-region level) analysis was conducted comparing the mean values of *T. gondii* prevalence and terrestrial watershed variables for each study region (electronic supplementary material, tables S1–S2). This analysis revealed strong associations between infection status and indices of land use and land cover change, particularly anthropogenic conversion.

A large range of prevalence values was recorded among regions. The overall mean sea otter *T. gondii* prevalence across all enrolled animals (*n* = 710) and study regions (*n* = 13) was 25.9%, but mean prevalence of study regions (electronic supplementary material, table S1) ranged from 70.6% (12/17) for otters sampled at Monterey Bay, California, to 0% at two sites in southeast Alaska (Whale Bay (*n* = 30) and Elfin Cove (*n* = 24)). The lowest prevalence observed anywhere in California was at San Nicholas island (4.2%). When comparing study regions by univariable logistic regression, two regions, both in California, exhibited statistically significant increases in prevalence when compared with the reference location of Big Sur, California—San Luis Obispo (OR = 3.89, *p* < 0.001) and Monterey Bay (OR = 9.48, *p* < 0.001).

Among-region differences in *T. gondii* prevalence were most strongly associated with human-dominated land uses and indices of human habitation (table 2). Cropping land (OR = 2.09, 95% confidence interval (CI) = 1.76–2.48) was the best predictor of sites with high *T. gondii* prevalence, but other anthropogenic land use metrics including combination of any human-modified land use, per cent impervious surface, and developed area and pasture were all significantly associated with high prevalence. Census-derived variables estimating road density, human population density and housing unit density were all associated with statistically significant increases in *T. gondii* prevalence risk, as were higher levels of scrub and wetland land cover. High levels of forest cover, by contrast, were associated with lower *T. gondii* prevalence than other land cover types (OR = 0.57, 95% CI = 0.45–0.73).

**Table 2.** Among-regions analysis. (Association between *T. gondii* prevalence among live captured sea otters and demographic and watershed variables assessed at study region level using univariable logistic regression on scaled and centred predictors. Includes entire study period (1998–2013) from Alaska, British Columbia, Washington and California. OR, odds ratio; 95% CI, 95% confidence interval; RD, road density; PD, population density; HD, housing density.)

predictor	estimate	OR	*p*-value	95% CI	AIC
cropping	0.74	2.09	0.0000	(1.76–2.48)	92.01
RD	1.02	2.78	0.0000	(2.08–3.73)	101.72
modified	0.87	2.39	0.0000	(1.88–3.04)	106.43
PD	0.64	1.89	0.0000	(1.57–2.27)	111.79
impervious	0.74	2.09	0.0000	(1.66–2.64)	120.26
developed	0.63	1.89	0.0000	(1.48–2.41)	135.16
HD	0.37	1.44	0.0000	(1.25–1.66)	136.34
pasture	0.47	1.61	0.0000	(1.34–1.93)	137.57
forest	−0.56	0.57	0.0000	(0.45–0.73)	142.07
scrub	0.53	1.69	0.0001	(1.29–2.21)	146.07
wetland	0.30	1.36	0.0076	(1.08–1.69)	156.68
other	−0.07	0.93	0.5459	(0.73–1.18)	163.18

### 2.3. Individual-level risk on a landscape scale

Combining approaches from the within- and among-region analyses, a landscape-scale individual analysis was conducted using estimated individual exposures to terrestrial variables, while also accounting for individual-level variables and using a random effect to control for between-region effects. This analysis found similar associations of land use to those seen in the among-regions analysis, even when using more precise individual-level exposure estimates. Prevalence of *T. gondii* infection across all study sites was significantly greater among male sea otters (31.8%) than females (23.3%; *p* = 0.017), and prevalence increased markedly with increasing age class (*p* = 0.005). In univariable logistic regression (electronic supplementary material, table S4), proximity to watersheds with increased forest cover was associated with lower than average *T. gondii* prevalence among sea otters (OR = 0.85, *p* < 0.001). Conversely, most other land cover types were associated with higher than average prevalence: the most statistically significant associations being with developed area (OR = 2.40, *p* < 0.001), grasslands (1.31, *p* < 0.001) and wetland area (OR = 1.96, *p* < 0.001). *Toxoplasma gondii* infection status was also associated with increasing human population density (OR = 1.19 per twofold increase, *p* < 0.001), housing unit density (OR = 1.20, *p* < 0.001) and road density (OR = 1.26, *p* < 0.001).

The final multivariate logistic regression model (table 3), including a random effect to account for dependence among samples collected in the same study region, found a highly significant (*p* < 0.001) association between prevalence and housing unit density, with 26% (*p* < 0.001) increase in infection odds associated with a doubling of housing unit density. In this multivariate model, increasing age (*p* = 0.001) and male sex (*p* < 0.001) were also associated with increasing prevalence. The model that included housing unit density was the best supported based on AIC comparison, although models substituting the other land cover variables associated with anthropogenic land use (high cropping land area, high developed area, low wetland area) also demonstrated significant associations with *T. gondii* prevalence.

**Table 3.** Individual-level landscape-scale analysis. (Multivariable mixed effects logistic regression model predicting *T. gondii* prevalence among live captured sea otters (1998–2013) from all study regions (including Alaska, British Columbia, Washington and California). A random effect is included to account for dependence of outcomes within study regions (*n* = 13). OR, odds ratio; 95% CI, 95% confidence interval.)

variable	level	OR	95% CI	*p*-value
sex	female	1.00	—	REF
	male	1.96	(1.28–2.97)	0.002
age	juvenile	1.00	—	REF
	subadult	7.83	(0.91–67.55)	0.061
	adult	29.60	(3.94–222.21)	0.001
housing unit density	twofold increase	1.26	(1.15–1.37)	0.000

## 3. Discussion

Sea otters have a broad distribution in the North Pacific, and so a correspondingly wide range of risk landscapes must be examined to fully understand all levels of drivers influencing the probability of *T. gondii* infection. Sea otter populations are structured at a fine spatial scale and home ranges are typically small [19,20], leading to great variation in individual exposures within a small area. Further, intrinsic and behavioural factors specific to each animal (prey choice in particular) can lead to niche partitioning, giving rise to heterogeneous disease risk among individuals—even among animals residing at the same coastal location [21]. Consequently, infection risk among individuals within a small area is largely associated with these individual-level risk factors that describe how an individual host interacts with its environment—specifically its consumption of a diet rich in invertebrates capable of concentrating *T. gondii* oocysts [22].

The among-region analysis demonstrated striking positive statistical associations of both human-modified land cover types (developed land, impervious surfaces, cropping and pasture) and human population density with *T. gondii* prevalence. This relationship persists when analysed using estimated individual exposures—the landscape-scale analysis on individual exposures showed significant associations with human-dominated land cover types, increasing human population density, increasing housing unit density and greater road density. Human population density and developed land uses are both associated with increased domestic cat density [13–15], and thus these associations are consistent with an important role of definitive host density in driving the large among-region differences in *T. gondii* prevalence. However, several other land-cover variables representing anthropogenic land use also showed associations with *T. gondii* prevalence, and more work will be needed to elucidate causal relationships between land cover and flow of pathogens from land to sea.

Spatial patterns observed in this study are consistent with an important role for oocyst loading (and hence domestic cat population density) and unobstructed runoff (via developed land, impervious surfaces, cropping land and pasture) underlying the observed associations with human housing unit density. Most of the very low prevalence sites are believed to contain fewer potential definitive hosts for *T. gondii* and are correspondingly less developed. The three Alaskan study regions, which exhibited 0% prevalence, were largely wild-land areas, probably holding very low densities of feral cats and few mountain lions (*Felis concolor*) [15,23]. Only one site in Alaska (WPWS) is within the distribution of lynx (*Lynx lynx*) [24]. San Nicholas Island sea otters (prevalence 4.2%) in this study were captured prior to the 2009–2012 feral cat eradication programme, but only 66 cats in total were removed, so the density of feral cats on this island must have been very low compared with mainland peri-urban areas [25] and no wild felids are known to live on San Nicholas Island. These results underscore the importance of definitive host density and terrestrial landscape features in *T. gondii* movement, consistent with existing theory that sea otters are infected by oocysts originating from terrestrial felids that find their way out to sea. As expected, infection does not appear to occur in sea otters in areas devoid of these definitive hosts. Regional variation either in felid population density or *T. gondii* shedding prevalence among wild or domestic felid definitive hosts have never been systematically examined, but it is possible that marked differences in these parameters between regions account for some of the observed variation. Though oocyst shedding prevalence has not been assessed outside of California, available evidence suggests felid population density is more important. While *T. gondii* prevalence is higher in wild than domestic felids in coastal California [12], domestic cats are far more abundant overall—VanWormer *et al*. [5] estimate that greater than 90% of felids actively shedding *T. gondii* along the central California coast are domestic cats. Both wild and domestic felids may serve as definitive hosts for *T. gondii*, and our results do show that infection occurs in areas with very few or no domestic cats; however, infection rates in these areas are lower than in populated areas of California where domestic cats are abundant. Thus, although Vancouver Island (British Columbia) is home to some of the densest populations of mountain lions in North America, sea otter *T. gondii* prevalences of only 13.3% (2/15) were recorded at the Clayoquot Sound study region, extending as far south as Tofino, BC and 3.3% (1/30) at the Nuchatlitz Inlet site, a more remote area of the west coast near the original sea otter reintroduction site.

Though there was a strong association between human housing unit density and *T. gondii* prevalence, marked variation in prevalence was observed among otters even in areas with low human population density, from 3.3% in British Columbia to 30% in Washington. A higher prevalence (97%) was reported for the same group of 30 animals from Washington in a prior study [26], however, these authors employed a modified agglutination test for *T. gondii* antibody detection that has not been validated for use in sea otters and performance of the two test methodologies has never been compared. The broad variation in prevalence at sparsely populated sites may be accounted for by differences in land cover variables that impact movement of oocysts across the landscape, including the potentially very important role of wetlands as filters [18]. Sea otter diets and other behavioural risk factors probably also differ among sites, as well as complexities of coastal hydrology not accounted for in this study [27].

The within-region analysis demonstrated the importance of examining risk factors at several spatial scales. When we examine infection risk over a small spatial extent, and include potential effects of individual-animal behaviour, we were able to uncover additional risk factors that were not apparent at the coarser scale. As noted previously [10], the dominant drivers of *T. gondii* infection risk were sex and prey choice, with animals consuming a diet rich in marine snails demonstrating markedly increased *T. gondii* prevalence. At this smaller spatial scale, consideration of human population density, housing unit density or indices of land cover did not provide additional predictive value. Although features of the terrestrial environment adjacent to sea otter habitat vary less over smaller spatial scales, marked differences were still apparent in land use and human population density within the study area, suggesting an important role for individual variation and small-scale processes in determining the realized infection risk for individual animals. Despite large-scale associations of *T. gondii* infection with dense human settlement, sampling otters residing offshore of more urbanized areas does not correspond to higher infection prevalence at this smaller scale; indeed, when two adjacent study regions, Monterey Peninsula and Big Sur were compared, a 33-fold difference in housing unit density was observed (electronic supplementary material, table S2), but only a modest difference (electronic supplementary material, table S1) in *T. gondii* prevalence (25% versus 20%). Individual risk factors appear to outweigh site-based differences in land cover at this scale, and small-scale processes not considered in the current analysis are also likely to affect the distribution of infection risk in the environment. The lack of a significant effect of land-cover variables at smaller scales may also be due in part to individual movements and ocean mixing that are more locally important in determining fine scale differences in sea otter exposures. Local-scale signal of land use influences on infection risk might be detectable in future studies if the non-uniform movement of oocysts out of outflows governed by topography, weather and ocean physical processes can be incorporated into models.

In this study, we applied a range of analytical scales to clarify environmental and demographic patterns for *T. gondii* infection risk for sea otters. We compared these results with those from a smaller number of tagged otters encompassing a smaller spatial scale, but with well-characterized behavioural and dietary history. Transmission risk for a given pathogen is a function of both the environment and how a host interacts with its environment. Each host exists in a risk landscape with areas of high and low pathogen exposure risk and each animal's infection risk is ultimately determined both by the regional risk landscape and the physiological and behavioural attributes of the host that determine how it interacts with its habitat. We expect that the combined forces of oocyst loading, mobilization across terrestrial surfaces into freshwater, wetland filtration, dispersal into ocean water, particle aggregation and invertebrate oocyst bio-concentration result in a complex and dynamic, three-dimensional risk matrix for sea otters that can vary through time, probably with an extremely low density of infectious particles in most areas, but with higher concentrations in certain locations and invertebrate prey items. Study design choices create the lens through which reality is translated into observations. Our current analysis has demonstrated that it is of critical importance to define mechanistically the elements of the transmission pathway targeted by any spatial analysis, and on this basis, choose an appropriate spatial scale, if accurate and meaningful conclusions are to be reached.

## 4. Material and methods

### 4.1. Data collection

**Figure 1.**
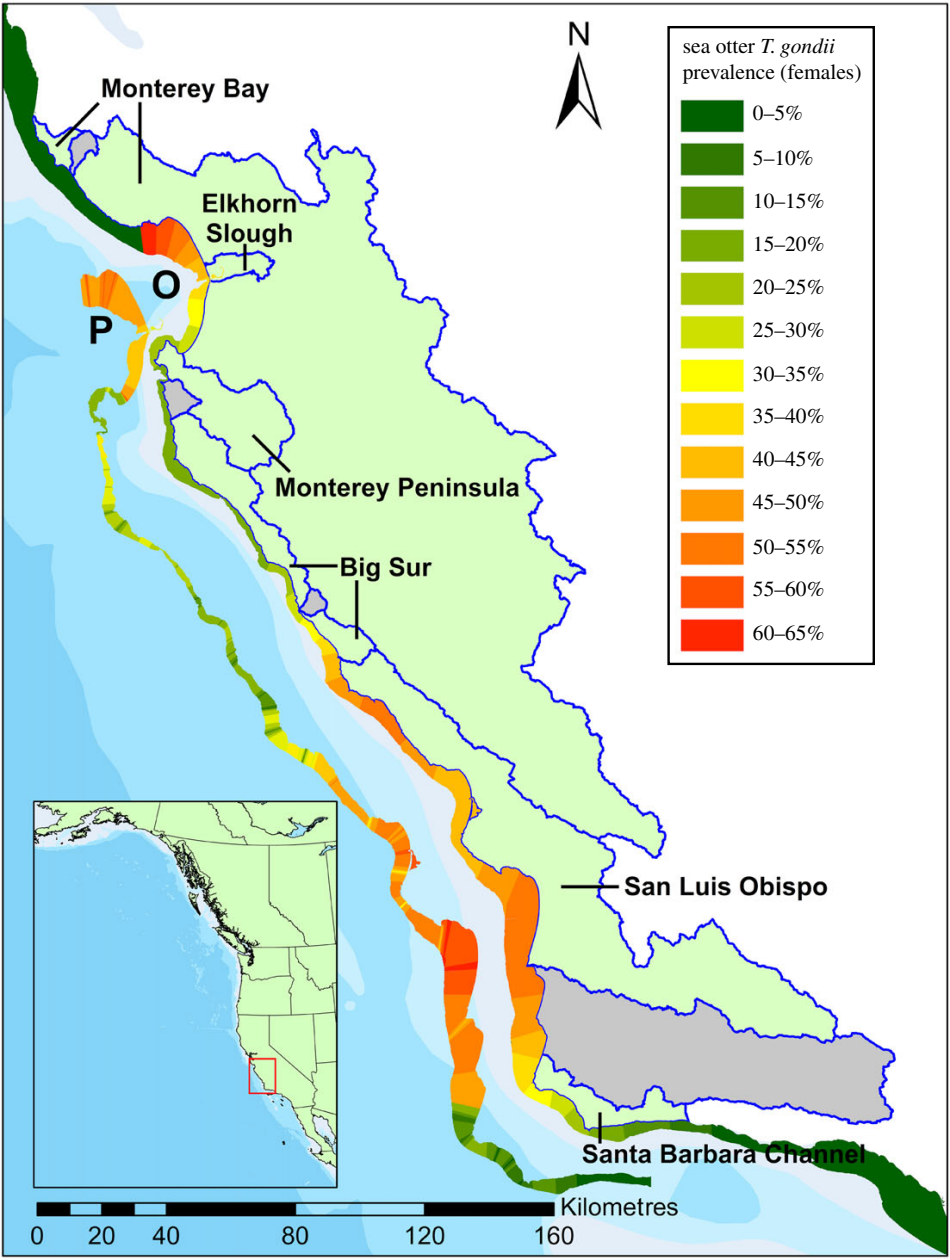
Map showing the six southern sea otter (*Enhydra lutris nereis*) study regions in mainland California (outlined in blue). Areas not included in this study are filled in grey. The multi-coloured bands show the extent of potential sea otter habitat (less than 30 m depth) in California, with colours indicating a smoothed estimate (generalized additive model; GAM) of the observed (O) *Toxoplasma gondii* infection prevalence (indirect fluorescent antibody test) for adult female sea otters and predicted (P) prevalence based on the final multivariate mixed effects model (individual-level risk on a landscape-scale - table 3), parametrized with the individually weighted terrestrial exposure values expected for an animal captured at that location. Data for females is displayed as female sea otters have smaller home ranges and greater site fidelity [19], and so their exposures are expected to more closely reflect their capture locations compared to males. See the electronic supplementary material, figure S1 for location of all northern sea otter (*E. lutris kenyoni*) study regions.

Sea otters (*n* = 710) were captured between 1999 and 2013 using Wilson traps operated by trained divers or with tangle nets [28] at 13 study regions consisting of between 1 and 509 coastal watersheds (electronic supplementary material, figure S1; figure 1). Animals were given a physical examination and anaesthetized using fentanyl and midazolam. Otters were flipper-tagged and blood samples were collected by jugular venipuncture. Biometric and demographic data (length, sex and weight) were recorded at the time of capture. Animals were classified into three age classes based on estimates of tooth wear—juvenile (0–1.5 years), subadult (1.5–3 years); and adult (greater than 3 years). Capture and sampling activities were covered by an institutional permit issued by the University of California, Santa Cruz, (Tinkt1306) and the Alaska Science Center (2010–9) and Federal Permits (MA672624, MA67925-2) issued by the U.S. Fish and Wildlife Service. Capture locations were recorded using a portable GPS device with a minimum precision level of 0.01′. In California, precise capture locations were not available for some study regions and captures conducted prior to 2003. For these captures, locations were manually geocoded to the nearest point, based on the ‘as the otter swims’ (ATOS) line, defined as a smoothed line of points following the 10 m isobath numbered in 0.5 km units from San Francisco Bay to the United States–Mexico border [29]. A subset of apparently healthy and not palpably pregnant animals (*n* = 131) were fitted at the time of capture with intra-abdominal VHF radio transmitters and archival time-depth recorders [30] and animals were then resighted by shore-based observers who recorded resight locations and prey composition (see Diet Analysis).

### 4.2. Sample collection and testing

Blood samples were collected via jugular venipuncture. Samples were allowed to clot and then were centrifuged at 1500*g* for 10 min. Serum was drawn off and stored at −70°C until tested for *T. gondii* antibodies using an immunofluorescent antibody test (IFAT), where a titer of ≥1 : 320 was regarded as positive. This test has been validated in sea otters and found to have a sensitivity of 96.4% and a specificity of 67.3% using a standard of current infection (as demonstrated by histopathology, parasite culture and immunohistochemistry) [9]. As such, a positive result is regarded as evidence of *T. gondii* infection, rather than merely past exposure.

### 4.3. Geospatial data

Hydrography for coastal watersheds contributing to study areas, including catchment boundaries, flow network and unique pour-points for each catchment were mapped using GIS modelling techniques with the medium resolution digital elevation datasets including the 10 m national elevation dataset (NED) and the 30 and 90 m Shuttle Radar Topography Mission (SRTM) [31,32]. Mean annual discharge (m^3^ s^−1^) and pollutant loads (kg yr^−1^) were modelled for each watershed (*n* = 746) with the Nonpoint Source Pollution and Erosion Comparison Tool (N-SPECT, NOAA Coastal Services Center) considering local topography, rainfall and land cover.

All watersheds with pour-points within a 21 km zone of influence from any sea otter capture were included in the dataset, as this distance encompasses 99% of individual dispersal distances over a 3-year period [19]. Discharge values were imputed using linear regression based on watershed area and all available discharge data for the small number of watersheds where estimates from this model were unavailable.

We collected publicly available data on features of the near shore environment hypothesized to influence *T. gondii* infection risk. Specifically, human population density, housing unit density, road density (as a proxy measure of human activity on the landscape) and the percentage of each land use class were calculated at the watershed-level using Geospatial Modelling Environment [33] and data from the US Census [34], Census of Canada [35], the National Land Cover Database [36] and North American Land Cover Database [37].

### 4.4. Individual exposure estimation

Individual-animal exposure to terrestrial features encompassed in the geospatial data was estimated based on capture location and the location of coastal catchment pour-points. Exposure of each enrolled otter to these variables, which are measured at the watershed level, was calculated by a weighting procedure, which accounts for both distance from animal capture location to pour-point and the amount of water discharged from the watershed. The exposure weighting (*W_i,j_*) for sea otter *i* to watershed *j* was calculated according to the following formula:

$$W_{i,j}\;=\;\frac{Q_{j}}{D_{ij}},$$

where *Q_j_* is the mean discharge (m^3^ s^−1^) of water from watershed *j* and *D_ij_* is the distance (km) between the capture location of sea otter *i* and the pour-point of watershed *j*. For captures in California (where the coastline is approximately linear, but some capture locations were manually geocoded as described above) distance was calculated along the ATOS line (see above) from capture location to the nearest pour-point location, yielding an ‘as the otter swims’ distance to the pour-point. Distances of between 0 and 250 m were rounded to 125 m. For study regions in Alaska, British Columbia and Washington, where coastlines are more complex, distances were calculated as the shortest path between capture location and pour-point with all land area coded as impenetrable barriers. Shortest paths were determined using the package ‘gdistance' in R [38] and path length was calculated using package ‘sp' on a universal transverse mercator (metres) projection. Animals were assumed to have no contact with watersheds more than 100 km from the capture location and the set of weightings for each sea otter were normalized to yield a sum of one.

The association between *T. gondii* serum antibody status and the resulting individually weighted exposure variables was analysed using mixed effects logistic regression models (see Statistical Analysis below). The final multivariate mixed effects regression model was used to predict *T. gondii* prevalence separately for adult males and females throughout California, based on the terrestrial features of California coastal watersheds. Prediction was not attempted in study areas outside California, owing to the low observed prevalence in these areas and small sample size collected at each site.

### 4.5. Diet analysis

Data on individual sea otter foraging behaviour were collected and analysed to estimate diet composition (proportion of consumed biomass represented by each prey type) using standardized methods described in previous studies [21,39]. Analyses were limited to animals with at 29 feeding dives recorded. Prey items were classified into 24 distinct functional groups based on taxonomy and morphology (electronic supplementary material, table S3) and biomass was calculated from prey counts and sizes [39,40].

### 4.6. Data analysis

In order to analyse variables associated with *T. gondii* infection at different spatial scales, three levels of analysis were employed: (i) *within-region analysis* employing fine-grained spatial data and incorporating individual-animal behavioural variables, restricted to a small spatial extent in central California; (ii) a large scale, coarse-grained *among*-*regions analysis* comparing infection prevalence to landscape variables aggregated at the regional level; and (iii) an *individual-level landscape-scale analysis* covering the same large extent as the among-regions analysis, but also employing the same fine-grained data on environmental variables as the within-region analysis in calculating individual-level independent variables.

Firstly, a *within-region analysis* was conducted on 131 tagged animals with sufficient observational data at three adjacent study regions in California (Monterey Bay, Monterey Peninsula and Big Sur). This analysis tested for individual and behavioural risk factors at the local scale. Since diet has previously been identified as an important risk factor for *T. gondii* infection [10], this analysis was restricted to animals with known diet histories based on behavioural observations. Ordinary logistic regression models were fitted to the data including adjustment for age and sex. Weighted exposures to watershed-level variables (based on capture locations, as described above) were included in the analysis to determine whether these variables were associated with prevalence at a small spatial scale.

Secondly an aggregated landscape-scale (*among-regions*) study of risk factors examined measures of terrestrial land cover and human population density. Associations between *T. gondii* prevalence in sea otters and study region average values of watershed-level variables (electronic supplementary material, table S2) were assessed using univariate binomial regression. Census-derived variables (human housing unit density, human population density and road density), and land use/land cover variables (urban/developed land, cropland, grazing land, forest, wetland and percentage impervious surface) were centred and scaled before inclusion in this analysis to aid comparison of results among models.

Finally, individual exposure estimation was used to assess *individual-level risk at a landscape scale*. This analysis aimed to determine whether an association between *T. gondii* infection status and terrestrial landscape features exists at the individual, rather than an aggregate level. In this step, individual-level variables (age and sex) were combined with estimated individual-level exposures to each landscape-scale variable (census-derived and land cover variables) were calculated (see *Individual Exposure Estimation*). Univariate logistic regression models were fitted using individual-level predictors and spatially weighted individual exposures to watershed-level variables. In order to reduce the false discovery rate only putative risk factors with *p* < 0.1 in the univariate analysis were used to build multivariable mixed effects models to assess risk factors for *T. gondii* infection status. A random effect of study region (*n* = 13) was included in all multivariate models to account for dependence among observations within the same study region. Multiple variables related to land use types and human population density were not included in the same multivariate model, as these variables were highly correlated. AICc was used to compare the degree of support for competing models. To produce a smoothed regional estimate of observed prevalence throughout the California coast, a binomial generalized additive model (GAM) was fitted to all prevalence data in California using sex as a binary predictor, and ATOS (along-shore) distance as a non-parametric smooth term. Smoothed estimates of prevalence and 95% CIs for the entire sea otter range in California were calculated from this model (figure 1).

## Supplementary Material

Figure S1: Study location map.

## Supplementary Material

Data tables

## Data accessibility

Detailed analytical results are available in the electronic supplementary material. Data are deposited at Dryad. http://dx.doi.org/10.5061/dryad.63s8h [41].
