# Supplementary material for: Defining the risk landscape in the context of pathogen pollution: Toxoplasma gondii in sea otters along the Pacific Rim
Source: R Soc Open Sci. 2018 Jul 4;5(7):171178. doi: 10.1098/rsos.171178 (PMC6083690; doi:10.1098/rsos.171178)
Supplement: Figure S1: Study location map. [file rsos171178supp1.pdf]

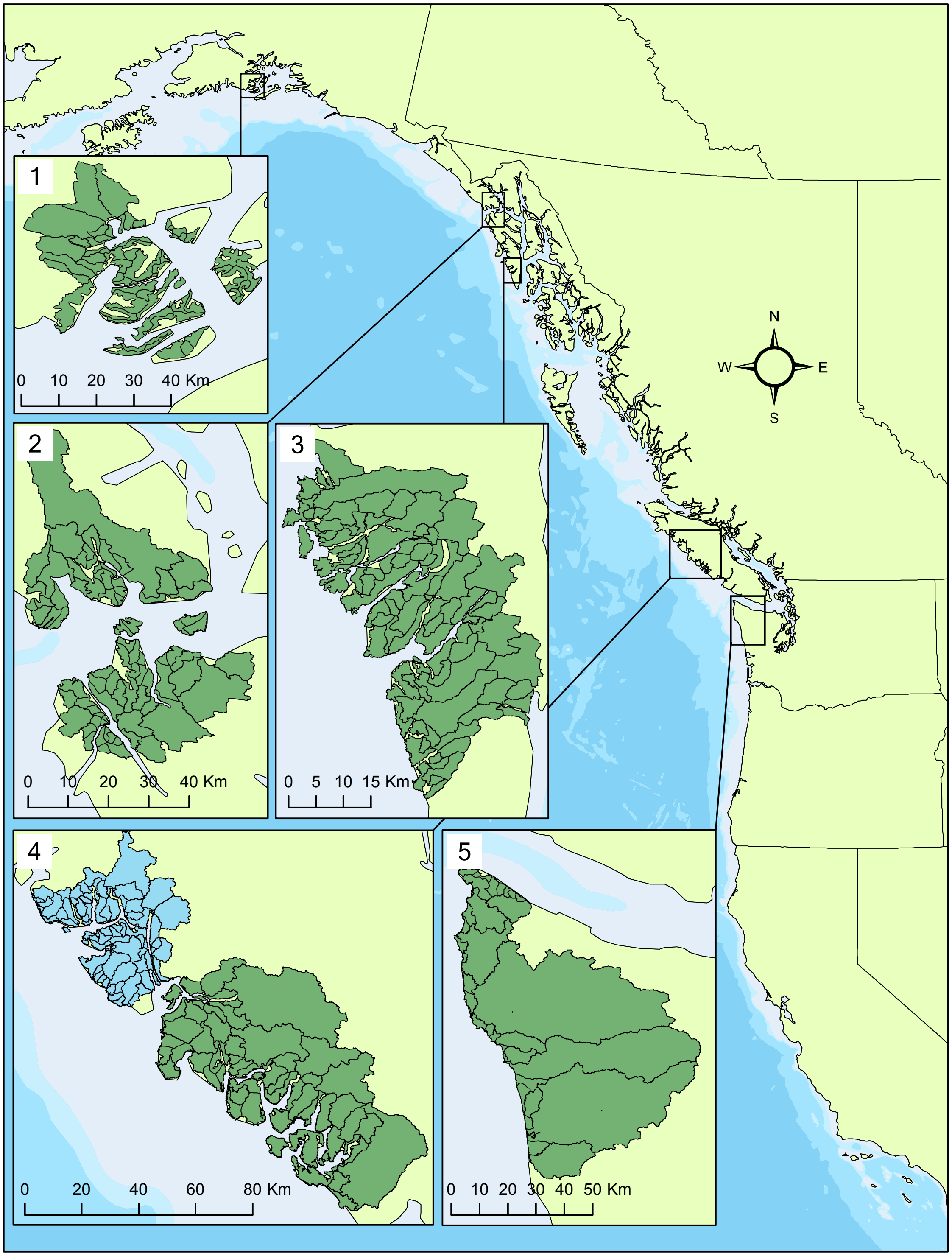

Figure S1: Map showing the location of all study regions for Northern sea otter (*Enhydra lutris kenyoni*). 1) Western Prince William Sound, Alaska; 2) Elfin Cove, Alaska; 3) Whale Bay, Alaska; 4) Nuchatlitz Inlet (Blue) and Clayoquot Sound (Green), British Columbia; 5) Olympic Peninsula, Washington. Coastal watersheds included in the study are outlined in black. See Figure 1 for Southern sea otter (*E. lutris nereis*) study regions in California.
