# Supplementary material for: Defining the risk landscape in the context of pathogen pollution: Toxoplasma gondii in sea otters along the Pacific Rim
Source: R Soc Open Sci. 2018 Jul 4;5(7):171178. doi: 10.1098/rsos.171178 (PMC6083690; doi:10.1098/rsos.171178)
Supplement: Data tables [file rsos171178supp2.pdf]

Table S1: Descriptive features of the study population. Prevalence of infection with *Toxoplasma gondii* is shown by age and sex, based on testing for antibodies in serum samples from live captured sea otters (1998-2013).

STUDY REGIONS	YEARS SAMPLED	SEX		AGE			PREVALENCE		
		F	M	Pup/Juv	Subadult	Adult	T-	T+	P
Western Prince William Sound, AK	2010	15	7	2	2	18	22	0	0.00%
Elfin Cove, AK	2011	19	5	0	7	17	24	0	0.00%
Whale Bay, AK	2011	24	6	1	0	29	30	0	0.00%
Nuchatlitz, BC	2010	17	13	0	1	29	29	1	3.30%
Clayoquot, BC	2010	10	5	0	3	12	13	2	13.30%
Olympic Peninsula, WA	2011	19	11	0	0	30	21	9	30.00%
Monterey Bay, CA	1998, 2001	6	11	0	2	15	5	12	70.60%
Elkhorn Slough, CA	2012, 2013	16	8	2	3	18	15	9	37.50%
Monterey Peninsula, CA	1998-2013	167	48	21	17	177	161	54	25.10%
Big Sur, CA	2003, 2008-2011	82	17	5	8	86	79	20	20.20%
San Luis Obispo, CA	2001-2003, 2005, 2012	85	56	8	18	115	71	70	49.60%
Santa Barbara Channel, CA	2012-2013	20	19	2	2	35	33	6	15.40%
San Nicholas Island, CA	2003-2005	10	14	2	5	17	23	1	4.20%

Table S2: Descriptive features of watershed variables by study region.

STUDY REGIONS	CENSUS VARIABLES				LANDUSE/LANDCOVER VARIABLES							
	Area (km^2)	PD	HD	RD	Developed	Forest	Pasture	Cropping	Wetland	Other	Modified	Impervious
Western Prince William Sound, AK	1032.9	0.07	0.06	0	0.00%	33.65%	0.00%	0.00%	1.70%	64.64%	0.00%	0.00%
Elfin Cove, AK	1841.2	0.06	0.07	0.01	0.03%	48.33%	0.00%	0.00%	1.55%	50.09%	0.03%	0.00%
Whale Bay, AK	1399	0.01	0.02	0	0.00%	48.69%	0.00%	0.00%	1.25%	50.06%	0.00%	0.00%
Nuchatlitz, BC	1437.2	0.05	0.01	0	0.00%	93.14%	0.00%	0.00%	0.00%	6.86%	0.00%	0.00%
Clayoquot, BC	3833.8	0	0	0	0.00%	89.95%	0.00%	0.00%	0.02%	10.03%	0.00%	0.00%
Olympic Peninsula, WA	4704.8	1.89	0.8	1.04	1.88%	79.96%	0.19%	0.01%	2.33%	15.63%	2.07%	0.31%
Monterey Bay, CA	13374.6	63.21	21.76	2.22	9.08%	21.53%	1.30%	8.79%	1.13%	58.17%	19.17%	1.76%
Elkhorn Slough, CA	132.1	101.18	30.13	3.16	29.29%	22.01%	0.89%	7.52%	5.34%	34.96%	37.69%	4.66%
Monterey Peninsula, CA	707.2	85.96	47.94	2.24	9.40%	48.85%	0.01%	0.19%	0.52%	41.02%	9.61%	1.81%
Big Sur, CA	764.6	2.23	1.43	1.23	2.79%	59.57%	0.09%	0.16%	0.33%	37.06%	3.05%	0.21%
San Luis Obispo, CA	3675	85.89	32.83	2.15	8.90%	22.18%	1.87%	7.27%	1.16%	58.63%	18.04%	2.09%
Santa Barbara Channel, CA	375.9	0.7	0.43	1.98	5.51%	25.33%	0.01%	0.29%	0.35%	68.52%	5.80%	0.61%
San Nicholas Island, CA	69.1	0.81	0.01	0.93	6.69%	0.01%	3.46%	0.00%	0.00%	89.84%	10.15%	1.58%

PD = Population Density, HD = Housing unit density, RD = road density (US Census 2010). Percentage of developed land, forest, pasture, cropping land, wetland, other and impervious surface are calculated from the National Land Cover Dataset (USGS) and North American land cover database (NRCan/CCRS). 'Modified' land is any human-dominated landscape, and is calculated here as the sum of developed, pasture, and cropping land.

Table S3: Quantitative diet analysis of 131 live captured sea otters from coastal California as a percentage of biomass.

Group	Urchin	Abalone	Mussel	Clam	Snail	Crab other	Kelp crab	Cancer crab	Sand crab	Star	Worm	Chiton	Sand dollar	Octopus	Squid	Misc. Rocky
Female	11.8%	12.6%	13.1%	5.8%	6.0%	5.6%	10.6%	20.4%	0.7%	1.3%	6.3%	0.9%	2.1%	0.9%	0.4%	1.4%
Male	5.5%	2.2%	9.1%	14.4%	13.4%	5.5%	4.2%	15.5%	2.9%	0.0%	17.4%	0.0%	7.7%	0.1%	1.8%	0.4%
Big Sur	9.7%	20.9%	15.8%	1.5%	6.9%	4.0%	8.6%	13.3%	1.5%	1.1%	10.7%	0.0%	2.4%	0.0%	0.0%	3.6%
Monterey Peninsula	10.9%	6.8%	11.1%	9.6%	7.5%	6.2%	9.8%	21.9%	1.0%	1.0%	7.5%	1.0%	3.4%	1.0%	0.9%	0.3%

Table S4: Landscape-scale Individual-level Analysis. Univariable logistic regression analysis predicting *Toxoplasma gondii* serum antibody status among live captured sea otters.

Variable	Level	OR	SE	95% CI	P
**Age**	Juvenile	1	-	-	REF
	Subadult	5.60	1.08	(0.67-46.46)	0.1105
	**Adult**	**17.38**	**1.02**	**(2.37-127.23)**	**0.0049**
**Length**	**Per 10 cm**	**1.17**	**0.05**	**(1.06-1.29)**	**0.0014**
**Sex**	Female	1	-	-	REF
	**Male**	**1.54**	**0.18**	**(1.08-2.19)**	**0.0166**
**Developed**	**10% increase**	**2.40**	**0.19**	**(1.66-3.48)**	**<0.0001**
**Row Crops**	**10% increase**	**1.98**	**0.24**	**(1.24-3.15)**	**0.0041**
Grazed Pasture	10% increase	2.72	1.11	(0.31-24.08)	0.3686
**Forest**	**10% increase**	**0.85**	**0.04**	**(0.78-0.93)**	**0.0003**
**Wetland**	**1% increase**	**1.96**	**0.14**	**(1.48-2.59)**	**<0.0001**
Scrub	10% increase	1.01	0.08	(0.87-1.18)	0.8805
**Grassland**	**10% increase**	**1.31**	**0.05**	**(1.2-1.44)**	**<0.0001**
**Other Natural Vegetation**	**10% increase**	**1.33**	**0.05**	**(1.2-1.47)**	**<0.0001**
**Impervious Surface**	**1% increase**	**1.34**	**0.08**	**(1.15-1.56)**	**0.0002**
**Human Population Density**	**2-fold increase**	**1.19**	**0.03**	**(1.13-1.25)**	**<0.0001**
**Human Housing Unit Density**	**2-fold increase**	**1.20**	**0.03**	**(1.14-1.27)**	**<0.0001**
**Road Density**	**2-fold increase**	**1.26**	**0.05**	**(1.15-1.38)**	**<0.0001**
**Study Area**	Big Sur, CA	1	-	-	REF
	Prince William Sound, AK	0.00	843.46	(0-Inf)	0.9847
	Elfin Cove, AK	0.00	807.55	(0-Inf)	0.9840
	Whale Bay, AK	0.00	722.30	(0-Inf)	0.9821
	Nuchatlitz, BC	0.14	1.05	(0.02-1.06)	0.0570
	Clayoquot, BC	0.61	0.80	(0.13-2.91)	0.5334
	Olympic Peninsula, WA	1.69	0.47	(0.67-4.26)	0.2632
	**Monterey Bay, CA**	**9.48**	**0.59**	**(2.99-30.03)**	**0.0001**
	Elkhorn Slough, CA	2.37	0.49	(0.91-6.2)	0.0784
	Monterey Peninsula, CA	1.32	0.30	(0.74-2.36)	0.3413
	**San Luis Obispo, CA**	**3.89**	**0.30**	**(2.16-7.03)**	**<0.0001**
	Santa Barbara Channel, CA	0.72	0.51	(0.26-1.95)	0.5159
	San Nicholas Island, CA	0.17	1.05	(0.02-1.35)	0.0939

Variables with P<0.10 were candidates for inclusion in the multivariable model. Includes entire study period (1998-2013) from Alaska, British Columbia, Washington and California

Table S5: Landscape-scale Individual-level Analysis. Model selection table including AIC_C values and weightings of multivariable mixed effects logistic regression models.

Variables	K	AICc	Delta_AICc	AICcWt	LL	Cum.Wt
SEX + AC + log2(HD)	6	688.88	0.00	0.715	-338.38	0.715
SEX + AC + Frm10 + log2(HD)	9	690.80	1.92	0.274	-338.32	0.989
SEX + AC + Wet1	8	698.15	9.27	0.007	-343.01	0.996
SEX + AC	6	701.52	12.64	0.001	-345.72	0.997
SEX + AC + Frm10	6	701.85	12.97	0.001	-344.86	0.998
SEX + AC + Dev10 * Frm10	6	702.52	13.64	0.001	-343.16	0.999
SEX + AC + Dev10	7	703.40	14.52	0.001	-345.64	1.000
SEX + AC + Dev10 + Frm10	5	703.83	14.95	0.000	-344.83	1.000

A random effect is included in all models to account for non-independence of outcomes within study regions (n=13). Higher weightings indicate greater support for the model based on how well they fit the data (AIC_C). SEX = sex (2 levels – Male, Female); AC = age class (3 levels – Pup/Juvenile, Subadult, Adult); log2(HD) = human housing unit density (housing units/km^2, $\log_2$-transform – i.e. per doubling of HU density); Frm10 = Farmland used for cropping (continuous - per 10% change in total watershed area); Dev10 = Developed land (continuous - per 10% change in total watershed area); Wet1 = Wetland area (continuous - per 1% change in total watershed area).
